# “So, when a woman becomes ill, the total structure of the family is affected, they can’t do anything…” Voices from the community on women with breast cancer in India: a qualitative focus group study

**DOI:** 10.1007/s00520-021-06475-4

**Published:** 2021-08-22

**Authors:** Sunitha Daniel, Chitra Venkateswaran, Charu Singh, Ann Hutchinson, Miriam J. Johnson

**Affiliations:** 1 General Hospital, Ernakulam, Kochi, India; 2 Wolfson Palliative Care Research Centre, University of Hull, Hull, HU6 7RX UK; 3 Department of Psychiatry, Palliative Care and Psycho Oncology, Believers Church Medical College, Thiruvalla, Kerala India; 4 Amrita Institute of Medical Sciences, Amrita Vishwa Vidyapeetham, Kochi, Kerala India

**Keywords:** Breast neoplasms; Psychological; Distress; Indian; Body image; Hair loss; Patriarchy; Family; Culture

## Abstract

**Background:**

Psychological symptoms are common in women with breast cancer and profoundly affect their role in the family and wider community, varying across cultural backgrounds. Breast cancer is becoming the most common cancer among women in India. We aimed to understand the cultural context within which Indian women with breast cancer living in India, experience psychological concerns from the perspectives of healthcare professionals, volunteers and church members.

**Methods:**

Five focus groups were conducted in South India (clinicians (2 groups)) lay public (3 groups). A topic guide was explored: understanding of breast cancer, experiences of patients with regard to diagnosis and treatment and psychological impact. Groups were audio-recorded and verbatim transcribed. Lay groups were conducted in Malayalam with translation and back-translation. Transcripts were subjected to thematic analysis using “cultural task analysis” as a lens for analysis.

**Results:**

Forty-five (oncologists (5), nurses (10), church members (16) and community volunteers working in a palliative care unit (14) participated. Three major themes psychosocial issues related to diagnosis, psychosocial impact of cancer treatment and coping with diagnosis and treatment and nine subthemes emerged from the two groups. All described psychological impact on women with breast cancer including body image, change of family role and their need for support. Family and faith were recognised as the major framework providing key support but also significant stress. Clinicians were also concerned about financial implications and issues around early cancer detection. Laypeople and nurses also commented that poor communication and lack of empathy from doctors aggravated distress.

**Conclusion:**

Clinical and lay communities were aware of the widespread psychological impact affecting women with breast cancer which are amplified by the patriarchal context within which they live, which extends into clinical practice. Family and faith provide a strong support structure and are a cause of distress, as core roles and expectations are challenged by this disease of womanhood.

## Background

Breast cancer is the most prevalent cancer among women globally. Psychological distress is common and is related to the impact of this disease on the woman’s role in the family and wider community [1–3]. A recent systematic review [2] found that Indian women with breast cancer, living in India or as immigrants in Canada experienced distress, both ameliorated and exacerbated by similar cultural issues. India is the world’s second-most populous country, with a rich culture including patriarchy, strong family systems and religious beliefs. The role of women in society has changed over time from a high position during the early Indus Valley civilisation [4, 5] to one of submissiveness to father, husband and son [6, 7]. A joint family system where several married couples and their children may live in the same house [8] has persisted and is further reinforced by urban-industrial civilisation [9]. Four major religions (Hinduism, Buddhism, Sikhism, Jainism) originated in India alongside indigenous faiths and tribal creeds which have survived for centuries. The influence of religion is embedded within the culture and “travels” with the person [2], influencing the responses and behaviours of Indian women anywhere in the world.

Breast cancer prevalence has been rising steadily in India, where it is currently the most common female cancer (25 to 31%) [10]. Unfortunately, most breast cancers are detected at a late stage; 50% are locally advanced at diagnosis. This is probably due to lack of awareness, a non-existent breast cancer screening programme, sociocultural barriers like the Purdah system and an unwillingness to expose themselves or subject themselves to breast examinations and imaging [11]. The primary modality of treatment is surgery, radiotherapy and chemotherapy, and is delivered through public sector hospitals or funded through private- and employer-funded insurance, personal out-of-pocket fees, community-based and non-profit organisations, and by external funds from loans and grants [12, 13]. A recent qualitative study among women with breast cancer [3] highlighted the psychological distress in Indian women in relation to prevailing societal attitudes regarding the role of women as wife, mother and the carer of older in-laws and hair loss as a particular source of distress. Greater support from the community and other social networks are shown to improve the quality of life of breast cancer patients after diagnosis [14] and their views will help us to understand the cultural context within which Indian women experience breast cancer, which is important for clinicians involved in their care.

We therefore explored this cultural context for Indian women living with breast cancer in one region in India from the perspectives of healthcare professionals, volunteers and church members.

## Methods

### Study design

We conducted a qualitative study using thematic analysis [15] as a theoretically flexible approach. Five focus groups (one group each of doctors, nurses and members of a local church’s women’s group and two groups of volunteers working with palliative care patients) gave data between October 2015 and January 2016. Doctors and nurses were selected to get an in-depth understanding of the perceptions of a healthcare worker with regard to breast cancer while experiences from laypeople were obtained from trained palliative care volunteers, who are part of neighbourhood network programme in palliative care [16]. A faith group is important because faith is such a structural influence in the state and the church group was selected as a convenience sample as it was the local church of the researcher (S.D.).

## Ethics

Institutional and ethical approvals (Research Ethics Committee, Hull York Medical School, UK) were gained prior to data collection.

### Setting

A single tertiary hospital, a single Christian church and a community palliative care unit are the volunteer group in the South Indian State of Kerala.

### Participants, invitation and sampling

Eligible participants who were able to give written informed consent were approached from (i) Department of Oncology, doctors and nurses; (ii) Department of Palliative Medicine, community-based volunteers and (iii) members of a local church’s women’s group.

Convenience samples for each group were obtained. Doctors were invited by S.D. and nurses by the Director of Nursing. Members of the church group were invited through the parish priest. Community volunteers were invited by palliative care staff. Participants gave written informed consent for audio-recording, use of anonymised quotes and use of data in future research.

### Data collection

Five semi-structured focus groups of approximately 45 min were conducted (S.D., with C.V., C.S.) using a topic guide to explore understanding of breast cancer, experiences of patients regarding diagnosis and treatment and psychological impact (Table 1).

**Table 1:** Focus group discussion topic guide

1) Could you please tell us more about your general understanding of breast cancer?
2) Would you like to share any experience of people diagnosed with breast cancer?
3) Could you please tell us more about your concepts of treatment experience?
4) Could you please talk about the psychological aspects of the disease and treatment?
5) Could you elaborate on your general understanding about difficulties patients could face?
6) Could you tell us more about your general ideas about role of women in family, role reversal, body image issues?
7) How do you think communities could support women with breast cancer?
8) Any further comments?

Clinicians’ groups were conducted in English and lay groups in Malayalam (regional language), recorded and transcribed. Those in Malayalam were translated into English (S.D.) and back-translated (C.V.). There were no study withdrawals.

### Data analysis

Transcripts and field notes were anonymised and checked for accuracy. NVivo 12 software was used to manage data. Data were subjected to thematic analysis [15] using “cultural task analysis” [17] as a lens, which describes how cultural nuances like patriarchy and the role of women in the family can affect how they experience the disease [18, 19] and make decisions with regard to treatment.

The following steps were conducted: (i) data familiarisation; (ii) line-by-line coding: S.D., C.V. and M.J. independently coded a group transcript and agreed to a code list, whilst allowing new codes to present; (iii) coding of all transcripts (S.D.); (iv) discussion (S.D., M.J.) to describe developing patterns of commonality (themes or convergence) and (v) agreement of analytic themes through further discussion (S.D., M.J.) ensuring distinct themes with consistent data [15]. Analysis was performed continuously; issues raised influenced subsequent group discussions. Data saturation was considered adequate when no new codes were emerging.

Analysis in English allowed the non-Indian researcher (M.J.) to participate helping to address potential challenges in reflexivity (S.D. and C.V. were Department physicians (palliative care, psychiatry) and both from the culture involved). M.J. is a UK clinical academic palliative physician.

## Results

### Participants

Characteristics of the 45 participants are summarised in Table 2.

**Table 2:** Sociodemographic characteristics of participants

Variable	Number
Age	Range of age (in years), median
	33–81, 50
Sex	Number
Male	10
Female	35
Number in each groups	
Oncologists	05
Nurses	10
Church members	16
Community volunteers	14
Education	Number
High school	13
Diploma	06
Graduate level	12
Post-graduate	07
Not known	07
Marital status	Number
Married	40
Single	02
Widow	01
Not known	02
Religion	Number
Hindu	11
Christian	29
Muslim	04
Indian	01
Number of years of experience for clinicians	Range in years, median
	9 to 35, 25

### Findings

Three major themes psychosocial issues related to diagnosis, psychosocial impact of cancer treatment and coping with diagnosis and treatment and seven subthemes emerged from the groups. We present the findings below with quotes given in Tables 3, 4 and 5 including other quotes supporting the results.

**Table 3:** Theme 1: Psychosocial issues related to diagnosis

Theme	Subtheme	Quotes
Psychosocial issues related to diagnosis	Recognition of psychological distress	Quote 1 “When disease is diagnosed, instead of seeking treatment, she will decide to keep the disease a secret, also that is a private part of my body, even my husband will reject me, such a fear. All this is part of a social stigma, what people have imposed on women saying a woman should be like this. The stigma in Indian culture is a major factor in this.” ( Volunteer2;3 Age 40–50) Quote 2 “There was a lady who was behaving abnormally at home, jumped into a well. It was a cancer in the curable stage, even then patient became like that, because of incomplete information. Then finally she got admitted and was managed when she became well enough to share her story she said’ don’t laugh I will tell you the reason, I was told I will lose my hair, when the doctor said like that I thought why should I live with no hair, my husband liked my hair and married me.” (she had a lot of hair) (Nurse1 Age 50–60) Quote 3 “tend to kind of prioritise and take care of the medical aspects and leave all the rest of it thinking that it will sort out on its own” (Doctor2/Male/40–50) Quote 4 “they think these issues are a part of life, because most of them, they are alone, their family goes out to work so there is no one to talk to them, family just ensures that they are well at home when they come back but do not ask how they feel when they are alone.” (Doctor 4 /Female 40–50) Quote 5 “surgeon can only answer in one word, a detailed conversation is not possible there, so when they ask “ Will I lose my hair doctor?’ They say yes, they can only answer that. But if they add one more sentence like ‘don’t worry it will come back’ it might be more reassuring to patient.”( Nurse1 Age 50–60) Quote 6 “Then she had chemotherapy, radiation, lost all her hair and then she became very upset mentally. All these are the things they say, lot of mental stress, losing hair so much, when they have their shower and see the hair falling like water, they can’t bear it.” (Church1 Age 60–70)
	Delayed diagnosis	Quote 7 “Generally breast cancer is something which, in most, maybe western countries it is screened and detected and screening is high but in India there is no organised screening.” (Doctor1 Male/50–60) Quote 8 “Now these things like mammography, just like when health workers used to collect samples from houses for filariasis etc., in a similar manner, with the support of the social leaders, government should make it compulsory that after 35 years, all women should do mammogram”( Volunteer1; 8 Age 40–50) Quote 9 “Today’s Indian woman is of the habit of living for others. She thinks my life is meant for others. so she will hide the disease thinking other will have to suffer for me.” (Volunteer2; 3Age 40–50) Quote 10 “Initially many people hide the disease. Only after it spreads and becomes unmanageable, it is communicated and then the situation will be beyond cure.” (Volunteer2; 1 Age 50–60) Quote 11 “because of that people who live in villages don’t know we can identify or do breast self-examination or the treatment and its complications, they don’t know anything about that” (Nurse 4 Age 40–50) Quote 12 “one of the biggest problems is stigma; many people are worried that if the news gets out, it is going to affect my daughter’s marriage, my family in general.” (Doctor 5/Male 50–60) Quote 13 “To avoid such treatment from expensive private hospitals, people take some effort and struggle but go to regional cancer centres to get treatment, even if the travel is so difficult, people tend to go like that. Because they know that expense will be too much if they go to nearby places”. (Church 5 Age 60–70) Quote 14 “her second breast was also diagnosed with the disease. So now though the doctor has told her chemotherapy was enough, she is not doing it. The single reason being, if she loses her hair again, the society will come to know of her disease again, that problem is haunting that family, she is ready to die but not ready to give up her hair…” (Volunteer2; 3 Age 40–50)

**Table 4:** Theme 2: Psychosocial impact of cancer treatment

Theme	Subtheme	Quotes
Psychosocial impact of cancer treatment	Experience of treatment	Quote 15 “The next thing is, of course surgery is usually the first line of treatment once the patient is diagnosed and I don’t think that it is either breast conservation or reconstruction is offered as often as it should be Quote 16 A lot of people are reluctant to come forward for surgery because of the fear of losing the breast. (Doctor1 Male/50–60) Quote 17 “our culture is totally different, or like somebody wearing a saree, it is not very obvious, she does not have one breast, I think it does not make much of a difference(Doctor 3/Female 50–60) Quote 18 “Most of the women feel great mental agony when breast is removed. Before surgery, all are given a psychological counselling now. That does some good. Some patients do plastic surgery after the removal surgery. But only those with money can afford that.” (Volunteer1; 8 Age 40–50) Quote 19 “I have known many who cannot reconcile with the removal of their breast. That is more prevalent among younger women around 30–35 years of age, than old women. Old women do not have that much difficulty.” (Volunteer1; 8 Age 40–50) Quote 20 “Also if it is working women financial issues also comes and job related issues so they will postpone the treatment till the job issues are sorted and settled”.” (Doctor 5/Male 50–60) Quote 21 “Post operatively patient develops lymph oedema; again this is due to lack of information. Lymphoedema is very disabling, they are free of disease for 8–10 years and they live with 15 kg of one limb”.(Nurse5 Age 60–70) Quote 22 “The society has taught all of us to consider long hair and breasts as the yardsticks of woman hood. So when you don’t have any of this, it is a very painful situation.” (Volunteer2; 3 Age 40–50) Quote 23 “If women have an illness, it is an issue from the start because that woman would have been the main person in the family, then there is a disruption of the rhythm in all aspects, taking care of children, husband, so it is very difficult.( Volunteer1; 5 Age 60–70) Quote 24 “So when they become ill, the total structure of the family is affected, they can’t do anything, so the kid’s studies will be affected, husband’s work is affected. Husband is not able to go to work, so the income of the family is affected. So the total structure of the family is getting changed. (Nurse1 Age 50–60) Quote 25 “In Kerala most women in house have an important role, right from getting up in the morning, cooking, getting kids ready for school, looking after husband, some husbands if they are lazy (laughing in background) right from getting their shirt ironed and ready to wear, feeding them, and getting their bags ready and giving it to them so that they can go for working”.(Nurse 10 Age 60–70)
	Experience of Medical care	Quote 26 “the hospital management said he is an elderly doctor, when he becomes busy with lot of patients he is not able to manage things and so gets angry.” (Church 8 Age 40–50)
		Quote 27 “To be quiet honest this is all a business to make money. Even when doctors know that the patient is going to die soon they still give radiation, chemotherapy do all treatment and get money.” (Church 14 Age 60–70)
		Quote 28 “Another experience is even if patients are diagnosed with cancer the doctors don’t behave in a compassionate manner with them. I know about an experience, if we ask anything to doctor he immediately loses temper and will not explain things to us. He would say ‘The patient is 80 years old, what more do you need, how long should she be alive?’” (Church8 Age 40–50)
		Quote 29 “Compared to doctors from other specialities those working in palliative care shows more love and compassion People working in palliative care are very caring and I have personal experience, they have at least a bit more compassion. We need more people like that.” (Church5 Age 60–70)
		Quote 30 “Here (public hospital) the treatment is very good. We do not have anything to complain. All the doctors and nurses, attenders, chemo ward staff, volunteers, are very good. I had gone to a private hospital before coming here. What I feel is, here we have better treatment than even that. All patients get equally good treatment. That government is doing all these things. Many people do not know. (Volunteer1; 8 Age 40–50)
	Solutions	Quote 31 “similarly, government should ensure adequate screening services for asymptomatic people then it will be more useful in detecting the disease.” ( Volunteer2; 1 Age 50–60) Quote 32 “I think what’s best you could do is probably just have people to provide inputs, have somebody who is always available on call to support, because all that they, I realize,, most of the time, twenty people who call, about ten or twelve of them just call for reassurance like this is nothing wrong, this is alright,” D5: (Doctor/Male 50–60) Quote 33 “How they are going to face the treatment, few days ahead, how you will face, what changes will happen to patient and what should you do?” (Nurse4 Age 40–50) Quote 34 “I still feel the best way to address it is to get people who have gone through it, show them the photograph, talk to them and make them talk. ‘I have also gone through this hair loss and my hair is grown back’. That confidence cannot come from any amount of preaching, it has to be actually talked to with the person who has gone through it.” (Doctor 5/Male 50–60) Quote 35 ‘Will I lose my hair doctor?’ They say yes, they can only answer that. But if they add one more sentence like ‘don’t worry it will come back’ it might be more reassuring to patient’. (Nurse 7 Age 40–50)

**Table 5:** Theme 3: Coping with diagnosis and treatment

Theme	Subtheme	Quotes
Coping with diagnosis and treatment	Response to illness	Quote 36 “encourage and support the patient to face the treatment bravely and help them to go through the illness.” They should create a supportive environment for the patient; all these are needed for the patient”. (Church 1 Age 60–70) Quote 37 “Also after staring chemotherapy we will really be fatigued, then we have to take rest and the whole family needs to support them, else they the patient would be low in mood and lose all hope. Also because of the mental strain they will continue to go downhill. If the family are very supportive then the patient would get better quickly”. (Church 9 Age70-80) Quote 38 “they don’t want to tell others they are cancer patients. ‘ how will public see me?’ I will lose all my hair’. (Nurse 5 Age 60–70) Quote 39 “There was an author and college lecturer who used to go to college and say, I am a cancer patient, keep an eye in newspaper for my obituary. So the students only knew about it when she spoke about that. Else she was not looking like a cancer patient.” (Nurse1 Age 50–60) Quote 40 “there was a patient who had breast cancer and had surgery, but her main concern was whether her husband would abandon her. As one of her breasts is removed she was concern that her husband would leave her. The patient used to talk to me about it and cry about that”. (Nurse 7 Age 40–50) Quote 41 “they become better, get their hair back, wear breast supports such things are available now, then they become better and cope.” (Church 1 Age 60–70) Quote 42 “Whereas there is another lady who is nearby, she after radiation, with no issues, went to temple with her husband daily, showing her power, for them, it will not come back again” (Volunteer1; 4 Age 40–50):
	Sources of support	Quote 43 “it is good for her children’s sake point of view”. Or “if she becomes depressed, same thing is going to affect children when they grow up if they ever have to face this again”.(Doctor 1 Male/50–60) Quote 44 “automatically become more religious, pray to God more” (Nurse 10 Age 60–70) Quote 45 “then socially like neighbours, people whom we interact with are all very compassionate to cancer patients, they provide them with whatever assistance they can. With all that these patients cope. Then they all have acceptance and they accept it as their fate.” (Nurse 3 Age 30–40) Quote 46 “Generally the Indian women sacrifice a lot for the family and they always keep their priorities the last. I had a lady whose husband had rectal cancer, she actually did not reveal about her breast lump till her husband completed his full treatment, only after that she told me that doctor I want you to look at my breast lump, then I realised that it was a large lump and we have lost almost 4–5 months.” (Doctor 4 /Female 40–50) Quote 47 “Sometimes they are worried if their husbands will leave them and go away they have to spend a lot of money for their treatment, or they are worried about their kids and them wasting a lot of money. “They have just started their life and they are spending a lot of money for my treatment”. “it is better to die’. There are people who say like that. (Nurse 4 Age 40–50) Quote 48 “Probably it is so much easier in western countries to voice out your love or concern which is probably still a taboo in our country. I don’t think there are too many husbands who go around saying, “no, don’t worry. I love you, I am still here.” I don’t do it, so I don’t know how the patient’s might be doing it”. (Doctor 2/Male/40–50) Quote 49 “People find solace in religion and spirituality. They look for prayer groups and attend that, they are consoled by people praying together. That will give them happiness. They will pray more.” (Church 8 Age 40–50) Quote 50 “I have a cousin, he wouldn’t even go to church but once the wife developed this condition, he started going to church, started praying for her, asked everyone to pray for his wife. Such husbands are also there”. (Church 5 Age 60–70) Quote 51 “Even if somehow the treatment is done and it is successful, she thinks, this organ is given to me by God, I should not allow the God given body to be cut and parts removed. That is a religious view” (Volunteer2; 3 Age 40–50):

#### Theme 1: Psychosocial issues related to diagnosis (Table 3)

- Recognition of psychological distress
  All groups felt that the psychological symptoms in women can start at diagnosis or any time during treatment and can be triggered by various factors including treatment-related body image changes on their sense of womanhood, fears of social stigma and thoughts of being abandoned by their spouse (quote 1)*.*
  These fears could affect behaviour and relationships, with sometimes extreme consequences (quote 2), aggravated by insufficient information or the opportunity to ask questions. Whilst some doctors acknowledged the need to address these symptoms and that they did not assess psychological distress systematically, most considered psychological distress would settle with tumour-related treatment alone (quote 3). If there were persistent psychological concerns, doctors felt unable to provide mental health support due to a perceived lack of time and an apparent choice to believe it is not their responsibility; these issues are just “part of their [patient’s] life” (quote 4). However, some commented that a dedicated team to provide psychological support or trained personnel to help them through the process of explaining the various modalities of treatment to the patient would help them address these issues better.
  Nurses acknowledged that short consultation times did not permit doctors to go into detailed support, but they felt doctors could improve their communication within their current resources. For example, one extra sentence, e.g. to reassure that hair would regrow following chemotherapy (quote 5) or to ask the nurse to arrange support from another patient, could help the patient significantly.
- Delayed diagnosis

Clinicians were concerned that cancer is usually detected at an advanced stage, due to the lack of universally accessible screening programmes (quote 7). This also concerned the lay groups who felt that such a screening system was a public health priority (quote 8). They had witnessed suffering due to late presentation and called for screening to be available, irrespective of ability to pay. Women also tended to ignore or hide their health needs because of reluctance to be examined by a male doctor and/or putting the needs of their families above their own (quote 9).

Stigma and false beliefs regarding cancer aetiology and diagnosis can stop women from seeking care. Most families still practiced arranged marriages and news of cancer in a family can seriously affect the marriage prospects of their daughters (quote 12). Delayed diagnosis may also be because the family is unable to afford the investigations or time away from work. Sometimes, they have to travel miles to attend an affordable cancer hospital, taking considerable time off work, thereby adding to their financial worries (quote 13). This can sometimes affect treatment decisions as women try to hide the news of cancer recurrence from the community by refusing treatment (quote 14).

#### Theme 2: Psychosocial impact of cancer treatment (Table 4)

- Experience of treatment
  There were concerns regarding the lack of national standard guidelines for treatment delivery (quote 15). The doctors felt that breast conservation may not be offered assuming that women are not bothered by how they look or the traditional Indian dress will cover any cosmetic disadvantage due to mastectomy (quote 17).
  However, the lay groups felt that breast removal does affect the women psychologically and highlighted the need for counselling (quote 18). They considered “long hair and breasts as the yardsticks of womanhood”. They also mentioned the crucial role of women in the Indian family and how there is a “disruption of the rhythm of family in all aspects”.
  Clinicians were very aware of and concerned about the financial impact of cancer treatment on the families of their patients (quote 20). Treatment side effects and body image issues are a major concern. Some patients develop lymphoedema, which the nurses felt could have been avoided/ameliorated by regular exercise and better post-operative care (quote 21), but which would carry additional costs for the family.
- Experience of medical care
  The lay groups expressed mixed opinions about the empathy and communication skills of the doctors that they have encountered. One member from the church group described how the “doctor gets angry when asked any questions” and complaints to the hospital management were apparently unaddressed (quote 26).
  Participants were very vocal about the financial gain of private hospitals, perceiving this to be to the detriment of patients (quote 27). They also reported lack of empathy and compassion from doctors except those working in palliative care (quote 29). The community-based volunteers worked with a public health palliative care unit felt that the government-funded hospitals had improved and were satisfied with the care they had witnessed (quote 30).
- Solutions

Note, solutions are also related to some aspects of themes 1 and 3, but are presented here. Solutions were suggested at a number of levels:

I. National: Screening programmes and public health education would address the public perception that breast cancer was usually incurable and encourage women to present earlier. Implementation of national cancer treatment guidelines would improve confidence that management was not primarily profit driven (quote 31).
II. Service: Increased resources to employ other clinical disciplines trained in mental health support such as social workers. Access to basic mental health and communication skills training for all clinicians and the development of peer support groups/buddy systems would also be helpful (quote 32). Inter-disciplinary team working, currently rare, would address some of the unhelpful role self-perceptions (e.g. “it’s not my job”) perpetuating unempathetic consultations by doctors.
III. Individuals: Timely, frank and honest conversations with the patients and family were needed. Lack of time was recognised as a challenge, but should not be seen as an excuse to abdicate responsibility. The need for doctors, in particular, to have an understanding of the impact of treatment side effects and to show empathy was highlighted. For example, acknowledgement of the distress due to hair loss and reassurance of re-growth would be a simple and non-time-consuming (quote 35).

#### Theme 3: Coping with diagnosis and treatment (Table 5)

- Response to illness
  Some women adapted to their different circumstances, finding ways to continue with their lives relatively undisturbed (quote 39). However, others were greatly affected by the disease and treatment. The distress caused by a cancer of such a key female organ, the distress related to breast surgery and its impact on the spousal relationship, in the context of the role of the Indian woman, was appreciated (quote 40).
- Sources of support

Family, children and motherhood were seen as a strong motivation, a means of coping and provided something to live for and the reason to undergo treatment (quote 43), with an implied obligation to undergo treatment for the sake of the family. In general, doctors viewed Indian women as self-sacrificing and nurturing mothers who ignore their own health while they look after the family. Faith and religion were seen as strong sources of support by all participants irrespective of their own religion, with an assumption that faith would make patients stronger and help them think positively (quote 44), though a thought was shared about a woman refusing surgery in view of “organ being given by God which cannot be cut” (quote 51) similar to faith fatalism.

However, some women feel unsupported by family when unable to carry out their expected role due to illness and feared spousal abandonment (quote 47). This may be complicated by cultural expression, or lack of expression, of emotion. Indian men rarely demonstrate emotions openly which may then be misinterpreted as being unsupportive (quote 48).

### Congruence and dissonance between professional and lay groups (Table 6)

**Table 6:** Areas of agreement, disagreement or silence between the groups

Subtheme	Doctors	Nurse	Community volunteers	Church members
Recognition of psychological distress	Agreement			
	All groups recognised psychological distress, and identified similar causes			
Delayed diagnosis	Agreement			
	All groups felt delayed diagnosis aggravated distress around diagnosis and fed erroneous public understanding about cancer, including surrounding stigma, which led to a vicious cycle of late presentation			
Experience of treatment	Disagreement			
	Acknowledged side effects could be distressing and sub-optimally managed Concerns about financial “toxicity” of treatment Suspicions that treatment options were driven by hospital profit and pharmaceutical companies rather than patient benefit Lack of national guidelines leading to variable treatment			
Experience of medical care	Agreement			
	There were uniform agreement about the care provided by palliative care team for symptom control, empathy and psychological support			
	Disagreement			
	Recognised care was disease focused and felt lacked skills and time to manage mental health	Doctors communicated poorly and could do better especially around expressing empathy regarding hair loss	Poor opinion of medical (doctor) care, particularly with regard to communication skills, information giving and being open to questions	
Solutions	Disagreement			
	More resources, healthcare workers with mental health training, e.g. social workers Better training and time in clinic	Better communication skills for the doctors even within resources, although agreed more resources and a system which valued person-centred care would be helpful	Further research is needed regarding the cause of the cancer, e.g. dietary issues	Cancer treatment should be free, which may also encourage early presentation
	Agreement			
	National screening programmes for all; national public education programmes; national clinical guidelines to manage the cancers; cancer should be a government priority			
Response to illness	Agreement			
	All groups agreed that the distress caused by the cancer of a female organ in relation to body image and disfigurement had profound effects on the relationship with spouse, sense of self and role. This was especially because of the importance placed on women as wife, lover, home-maker, mother and carer of the elderly in the Indian culture			
	Disagreement			
	Doctors felt that the impact of loss of breast, etc. was less of an issue for Indian women because of national dress	Nurses highlighted the impact of lymphoedema especially, which is not hidden by national dress	Lay groups both felt that loss of the breast was felt as keenly as in Western cultures, if not more so, by Indian women, given the importance of her almost sole role in the family	
Sources of support	Agreement			
	Family and faith were considered as strong sources of support by all groups However, all also could see that the family and faith centred society was a cause of distress in some, particularly when faith practices were disrupted, and family expectations not met and families did not accept the women’s needs, or where interpretations of faith fed into erroneous health beliefs and stigma			

In general, professional and lay groups agreed that psychological distress was significant, affecting treatment choices and responses to the disease. Both lay groups identified the vicious cycle of late presentation and stigma associated with erroneous health beliefs as being aggravated by the lack of national screening and public education programmes. All groups were quick to identify family and faith as strong sources of support. However, all admitted that these could also cause significant distress.

The main differences between the professional and lay groups centred on responsibilities and solutions. The doctors recognised the distress although, apart from some exceptions, did not address this, denying responsibility, skills or time. Their solutions were more time and additional trained staff. Other participants felt that the doctor did have an important role in providing empathic communication, and nurses felt that even small changes within resources would make a big difference to patients. Lay groups were suspicious of financial motivation for treatment, with poor confidence that treatment provided was standard, aggravated by poor information giving by doctors. However, clinicians were themselves distressed at the financial burden placed on patients and their families by treatment costs.

## Discussion

When viewed through the lens of the “cultural task analysis” [17], the cultural nuances within which an Indian woman experiences her breast cancer strongly affect her psychological experience. This was particularly seen in relation to patriarchy, also visible within the clinical teams; the role of women and family and faith. Significant distress affected how women presented with and responded to breast cancer, accessed support and navigated family role changes. A vicious cycle of erroneous health beliefs, late presentation and a lack of national screening programme was identified. Family and faith were considered a major support by all, but the impact of breast cancer on a woman’s primary role in the home was seen as particularly hard, adversely affecting the whole family. Therefore, although the family provided support, it also caused distress if the woman, or those around her, felt she was unable to fulfil her priority role, aggravated by a lack of displays of reassuring affection from male relatives. A range of solutions was suggested, from national programmes of education and screening to better care—especially regarding empathic clinical communication skills, and local peer “buddy” support. Nurses and lay groups felt doctors could communicate better even within time constraints, but doctors—good examples of care notwithstanding—were constrained by a bio-medical focus, lack of time and other team resources, e.g. social workers, appearing to choose to view the women’s distress as the women’s responsibility.

Diagnostic delay and late presentation (about half of newly diagnosed breast cancers in India [20, 21]) contribute to a worse prognosis and a belief that breast cancer is incurable. This fuels delayed presentation and diagnosis. The lack of an organised screening programme [22] was seen as a key factor in this vicious cycle. Indian women, particularly older women [23], despite reporting poorer health than men, have lower rates of healthcare utilisation, with fewer hospitalisation and outpatient encounters [24]. Indian women living in rural and peri-urban neighbourhoods have poor knowledge of breast cancer, with a minority practicing self-examination and none presenting for clinical breast examination [25]. Immigrant Indian women living in the West who are more educated and acculturated appear to engage better with the breast cancer screening services [26, 27]. National programmes to increase awareness with affordable (free to patient) screening would foster early presentation.

Stigma was highlighted by all, contributing to delayed diagnosis, affecting how women coped with the disease, affected treatment decision and from other work, a lack of active involvement during medical encounters or decision-making [28]. Women usually have arranged marriages and have repeated reminders of the importance of the birth of a first child within the first year of marriage [29]. These philosophies can affect the health-seeking behaviour of women, with some refusing treatment because of fears of infertility and stigma, with hair loss seen as a “hallmark” of cancer. Women appear to be particularly vulnerable to social stigma and seem to be a factor in treatment delay in other conditions like tuberculosis [30].

The patriarchal culture influences the way women respond to illness [31]. This is consistent with other reports from patients from a similar cultural background [3]. Incomplete information and the lack of opportunity to ask questions have been noted; women are usually accompanied by a “male carer who did all the talking” and remained passive during consultations [28]. Exclusion from decision-making was a cause of psychological distress of Indian women in Canada [32], exacerbated by language barriers. Our doctor participants considered that a well-informed patient coped better, but prevailing attitudes keeping the doctor at top of a hierarchy, seems difficult to change and even suggestions from nurses (often women) similar to ones from our study regarding communication may neither be given nor received easily.

The collectivist family [33] was considered as strong sources of support. However, consistent with our previous work [2, 3], a “two-edged sword” was described whereby some women felt “unsupported” and worried particularly about “spousal abandonment”, aggravated by a cultural reluctance of Indian men to show emotion. Similarly, faith practice is described as a major source of strength and resilience consistent in various other cultures [34, 35]. However, as with our previous work, issues in relation to faith can also be a cause of distress [2, 3].

Our study gives insight into the experiences in both the public and private healthcare sector; our clinicians were employed in a private institution; volunteers were attached to a government community team; church members had a mixed experience but mainly accessed private care. Church group members raised concerns about the influence of financial drivers for institutions, aggravated by the lack of national treatment guidelines, and the poor quality of medical care regarding compassionate communication and information giving. Reports of corrupt and irrational practices including referrals for unnecessary investigations and practice of non-evidence-based treatment for commercial gain are widely known in medical circles, but public dialogue on this is lacking in India [36, 37]. Another study of clinicians’ perspectives described the medical encounter as both “authoritarian” and “consumerist”, although power incongruities in the doctor-patient relationship are variable and subject to change [28]. However, there were pockets of good practice in our clinician’s data as shown from the study of women with breast cancer receiving care from the same private hospital as the clinicians in our study [3]. They reported mainly good, compassionate care from their oncologists, but found this surprising, and in contrast to previous poor medical encounters [3].

The need for better communication, particularly by doctors, was emphasised by all. This appears to be both an individual and system issue, with no priority given at the institutional level to communication skills training, or inclusion of suitably skilled team members (such as social workers). Cultural differences in communication are recognised and the paternalistic, hierarchical communication style in South East Asia is common, but not well received by patients [38]. Poor communication is reported elsewhere, for example, in chronic non-communicable disease management clinics [39]. Communication skills training is recognised as necessary for clinicians but there is no structured programme and limited availability [40]. Lack of effective communication and poor doctor-patient relationships lead to patient and carer dissatisfaction and are associated with litigation [41], and even violence against doctors in the country [42, 43]. Consultation models [44] which cater to disclosure of concerns, ideas and expectations by patients should be a part of “gathering information” within the communication framework and exemplify a patient-centred approach [45]. In response to a universal call for a change, the undergraduate medical training in the country has recently been amended to a competency-based curriculum with the aim to introduce the Attitude, Ethics and Communication module (AETCOM) as a longitudinal programme over the duration of the course [46]. A similar nationwide approach to post-graduate training would build on this, but issues around professional identity culture and funding priorities at individual institutions would need to be addressed. Of note, none of our participants mentioned communication skills training as a solution.

### Strengths and limitations

This is the first qualitative study from India to explore the understanding of clinicians and non-clinicians on the topic, enabling interpretation of clinicians’ views in the light of the lay public opinion. We captured experiences of public and private healthcare settings, from both upper and lower socio-economic groups. These data will not be, and are not intended to be, representative of the whole South Asian population. However, our findings are likely to be applicable to Indian women elsewhere, such as the experiences of accessing medical care [2], and the principle need to be cognisant of the woman’s cultural context when providing breast cancer care can be extrapolated to the rest of the country and to migrant Indian women who often “take their culture with them” [2]. Lay volunteers have some clinical experience and so will not be totally representative of lay voices with no experience; however, this experience is firmly rooted in the community rather than in the hospital. The inclusion of only one Christian church is a limitation; however, Kerala State is recognised for its inter-faith harmony and understanding of other faith cultures. Furthermore, the participants were almost all women; indeed, this study aimed to highlight the experience of women, in a culture where this voice is typically less prominent. However, although men were represented in both professional and lay groups, we recognise that we have fewer direct accounts of men’s experiences. Of note, men were invited to the church group as well as women, but only women attended. Although this may indicate that the topic is viewed as a “women’s problem only”, the data we generated clearly indicated how this “woman’s problem” affected the whole of family life and society. The experience of breast cancer patients was reported by individuals who did not experience the illness themselves.

### Clinical and research implications

A call for a screening programme and public education, particularly in rural communities, was voiced, surely a priority for this prevalent cancer. Post-graduate communication skills training for doctors and nurses, prioritised by institutions, would help address a lack of effective empathic communication. A greater emphasis and understanding of multi-disciplinary team working, with resources to include social workers, are needed. Culturally tailored community support groups and/or patient “buddy” systems can provide women with psychological and practical help.

## Conclusions

Indian women with breast cancer experience widespread psychological impacts. These are amplified by the patriarchal context within which they live, which extends into clinical practice. Family and faith provide a strong support structure and are a cause of distress, as core roles and expectations are challenged by this disease of womanhood. Poor education, late presentation and lack of universal screening lead to a vicious cycle of stigma aggravating distress. These findings are important for all clinicians caring for Indian women with breast cancer.

## Data Availability

Requests by authorised researchers for access to anonymised data may be made to the corresponding authors.

## References

[1] Luutonen S (2011). Breast cancer patients receiving postoperative radiotherapy: distress, depressive symptoms and unmet needs of psychosocial support. Radiother Oncol.

[2] Daniel S, et al. (2020) Psychological concerns of Indian women with breast cancer in different national contexts: a systematic review and mixed-methods synthesis. BMJ Support Palliat Care. Online first 10.1136/bmjspcare-2019-002076 32393530

[3] Daniel S, et al. (2020) ‘I don’t talk about my distress to others; I feel that I have to suffer my problems...’Voices of Indian women with breast cancer: a qualitative interview study. Support Care Cancer, p. 1–10 10.1007/s00520-020-05756-8 PMC7981292 32955655

[4] Fisher MH (2018) An environmental history of India: from earliest times to the twenty-first century. Vol. 18: Cambridge University Press

[5] Kapur P (1974) The changing status of the working woman in India. Vikas Publishing House New Delhi

[6] Robb P (2011) A history of India. Macmillan International Higher Education

[7] Ramusack BN, Sievers S, Sievers SL (1999) Women in Asia: restoring women to history. Indiana University Press

[8] David GM (1948). The family in India. Southwest J Anthropol.

[9] Mullaiti L (1995). Families in India: beliefs and realities. J Comp Fam Stud.

[10] Statistics of Breast Cancer in India: Trends in India. 2017 [cited 2017 12/12/17]; Available from: http://www.breastcancerindia.net/statistics/trends.html

[11] Gaurav A (2007). Spectrum of breast cancer in Asian women. World J Surg.

[12] Desai PB (2002) Cancer control efforts in the Indian subcontinent. Jpn J Clin Oncol 32(suppl_1): p. S13-S16 10.1093/jjco/hye139 11959872

[13] Moore MA (2010). Cancer epidemiology in South Asia-past, present and future. Asian Pac J Cancer Prev.

[14] Kroenke CH (2013). Social networks, social support mechanisms, and quality of life after breast cancer diagnosis. Breast Cancer Res Treat.

[15] Braun V, Clarke V (2006). Using thematic analysis in psychology. Qual Res Psychol.

[16] Sallnow L, Kumar S, Numpeli M (2010). Home-based palliative care in Kerala, India: the neighbourhood network in palliative care. Prog Palliat Care.

[17] Kitayama S (2009). A cultural task analysis of implicit independence: comparing North America, Western Europe, and East Asia. J Pers Soc Psychol.

[18] Alden DL (2014). Cultural targeting and tailoring of shared decision making technology: a theoretical framework for improving the effectiveness of patient decision aids in culturally diverse groups. Soc Sci Med.

[19] Kitayama S, Imada T (2010) Implicit independence and interdependence. B. Mesquita, LF Barrett, ER Smith, The mind in context, p. 174–200

[20] Akhtar M (2011). Is locally advanced breast cancer a neglected disease?. Indian J Cancer.

[21] Chopra R (2001). The Indian scene. J Clin Oncol: Off J Am Soc Clin Oncol.

[22] Rizwan M, Saadullah M (2009). Lack of awareness about breast cancer and its screening in developing countries. Indian J Cancer.

[23] Fikree FF, Pasha O (2004). Role of gender in health disparity: the South Asian context. BMJ.

[24] Roy K, Chaudhuri A (2008). Influence of socioeconomic status, wealth and financial empowerment on gender differences in health and healthcare utilization in later life: evidence from India. Soc Sci Med.

[25] Sharma PK, et al. (2013) Knowledge, attitude and preventive practices of South Indian women towards breast cancer. Health, 1(1)

[26] Sadler GR (2001). Asian Indian women: knowledge, attitudes and behaviors toward breast cancer early detection. Public Health Nurs.

[27] Choudhry U, Srivastava R, Fitch MI (1998) Breast cancer detection practices of south Asian women: knowledge, attitudes, and beliefs. in Oncology Nursing Forum 9826837

[28] Fochsen G, Deshpande K, Thorson A (2006). Power imbalance and consumerism in the doctor-patient relationship: health care providers’ experiences of patient encounters in a rural district in India. Qual Health Res.

[29] Fisher JA, Bowman M, Thomas T (2003). Issues for South Asian Indian patients surrounding sexuality, fertility, and childbirth in the US health care system. J Am Board Fam Pract.

[30] Nair DM, George A, Chacko K (1997). Tuberculosis in Bombay: new insights from poor urban patients. Health Policy Plan.

[31] Chekki DA (1988). Recent directions in family research: India and North America. J Comp Fam Stud.

[32] Gurm BK (2008). Understanding Canadian Punjabi-speaking South Asian women’s experience of breast cancer: a qualitative study. Int J Nurs Stud.

[33] Chadda RK, Deb KS (2013). Indian family systems, collectivistic society and psychotherapy. Indian J Psychiatr.

[34] Ashing-Giwa KT (2004). Understanding the breast cancer experience of women: a qualitative study of African American, Asian American Latina and Caucasian cancer survivors. Psychooncology.

[35] Puchalski CM (2012) Spirituality in the cancer trajectory. Ann Oncol 23(suppl_3): p. 49–55. 10.1093/annonc/mds088 22628416

[36] Patel V (2015). Assuring health coverage for all in India. The Lancet.

[37] Kay M (2015) The unethical revenue targets that India’s corporate hospitals set their doctors. BMJ 351: p. h4312 10.1136/bmj.h4312 26337575

[38] Claramita M (2013). Doctor–patient communication in Southeast Asia: a different culture?. Adv Health Sci Educ.

[39] Humphries C, et al. (2019) Patient and healthcare provider knowledge, attitudes and barriers to handover and healthcare communication during chronic disease inpatient care in India: a qualitative exploratory study. BMJ open, 9(11) 10.1136/bmjopen-2018-028199 PMC6858202 31719070

[40] Chatterjee S, Choudhury N (2011). Medical communication skills training in the Indian setting: Need of the hour. Asian J Transfus Sci.

[41] Abdelrahman W, Abdelmageed A (2017) Understanding patient complaints. BMJ. 356

[42] Nagpal N (2017). Incidents of violence against doctors in India: can these be prevented?. Natl Med J India.

[43] Kar SP (2017). Addressing underlying causes of violence against doctors in India. The Lancet.

[44] Denness C (2013). What are consultation models for?. InnovAit.

[45] Matthys J (2009). Patients’ ideas, concerns, and expectations (ICE) in general practice: impact on prescribing. Br J Gen Pract.

[46] Medical Council of India. Attitude, Ethics and Communication (AETCOM) Competencies for the Indian Medical Graduate 2018 2017 [cited 2020 September 20]; Available from: https://www.mciindia.org/CMS/information-desk/for-colleges/ug-curriculum.

